# Perceptions of oral nicotine pouches & their marketing among Ohio Appalachia smokers and smokeless tobacco users

**DOI:** 10.1371/journal.pone.0293597

**Published:** 2023-10-30

**Authors:** Lauren Long, Mahmood A. Alalwan, Brittney Keller-Hamilton, Joanne G. Patterson, Megan E. Roberts, Theodore L. Wagener, Leanne Atkinson, Sriya Suraapaneni, Darren Mays

**Affiliations:** 1 Department of Internal Medicine, The Ohio State University College of Medicine, Center for Tobacco Research, The Ohio State University Comprehensive Cancer Center, Columbus, Ohio, United States of America; 2 Division of Health Behavior and Health Promotion, College of Public Health, The Ohio State University, Columbus, Ohio, United States of America; University of Lincoln College of Science, UNITED KINGDOM

## Abstract

**Background:**

Oral nicotine pouches (ONPs) are novel products, gaining popularity and marketed as “tobacco-free” alternatives to cigarettes and smokeless tobacco (SLT), but their public health impact is unknown. This study qualitatively examined ONP appeal and perceptions among cigarette smokers and SLT users from Ohio Appalachia.

**Methods:**

In 2022, we conducted 10 virtual focus groups with smokers (n = 19) and smokeless tobacco users (n = 18) from Appalachia Ohio aged ≥21 to examine perceptions of risks and benefits, substitutability for cigarettes and SLT, and ONP marketing. We transcribed focus groups verbatim, thematically coded transcripts, and analyzed coded data for prominent themes.

**Results:**

Participants perceived ONPs to have similar or less risk than cigarettes/SLT but prominently discussed gastrointestinal and cardiovascular risks. Addiction risk was thought to be comparable to cigarettes/SLT, citing “nicotine is nicotine.” Participants viewed ONPs to be situational rather than complete substitutes for cigarettes/SLT, viewing them as “cleaner,” more socially acceptable, and discrete. Despite appealing features of ONP marketing, participants surmised ads would appeal to youth, new users, tobacco users seeking to cut down/quit, or to “high class,” “white-collar” demographics.

**Conclusions:**

Participants’ perceptions of ONPs and their marketing suggest ONPs are more likely to be used as situational versus complete substitutes for cigarettes and SLT. While situational substitution could exacerbate disparities in Appalachia by facilitating more frequent tobacco/nicotine use, complete substitution could reduce disparities. Research is needed to understand how perceptions, the appeal of ONP marketing, and novel product features translate to patterns of use to understand ONPs’ potential impact.

## Introduction

Cigarette and smokeless tobacco (SLT, i.e., chew, snuff, dip) use are more prevalent in Appalachia than the general population and declines in tobacco use in Appalachia have not matched the U.S. overall [1], widening health disparities [2–4]. Tobacco use disparities in Appalachia are due in part to social norms supporting use in the home [5], viewing tobacco use as a rite of passage [6, 7], and economic dependence on tobacco farming [5]. Further, tobacco companies have promoted SLT as an alternative to cigarette smoking in situations where it is unsafe to smoke (e.g., coal mining) [8], used marketing reinforcing Appalachian cultural values [6, 9], and systematically manipulated products to maximize their addictiveness [10, 11]. As tobacco companies introduce novel products, it is critical to understand these products’ appeal and how they may impact cigarette and SLT use in Appalachia.

Oral nicotine pouches (ONPs) are novel products that are available in 2 to >10 mg nicotine per pouch and in a variety of flavors (e.g., mint, fruit, coffee). ONPs come in containers with 15 to 20 pouches containing nicotine, flavoring, fillers, and other ingredients. ONPs are placed between the upper lip and gum to deliver nicotine, but they do not contain tobacco or require spitting [12]. ONPs became available nationally in 2019, and since then brands have rapidly grown and sales have surged [12–14]. The limited available evidence suggests interest in using ONPs and ONP trial is highest among adults who use combustible (e.g., cigarettes) and non-combustible (e.g., SLT) tobacco, but they may appeal to young, non-tobacco users as well [15–17].

Research to date on the potential health and addiction risks of ONPs has primarily been conducted by tobacco companies, and independent evaluations are needed. However, the available evidence suggests ONPs may expose adult tobacco users to fewer toxicants than cigarettes or SLT, including heavy metals, tobacco-specific nitrosamines, carbonyls, and other harmful and potentially harmful chemicals [18] The available evidence also indicates that ONPs have similar nicotine pharmacokinetics (i.e., deliver nicotine to users in similar amounts and with similar speed) to SLT and nicotine gum [19]. Marketing portrays ONPs as “tobacco-free” alternatives to cigarettes and SLT and conveys features such as nicotine content and flavor [12, 20]. However, since ONPs are new there is little research on how tobacco users perceive ONPs and ONP marketing. This is critical to begin to understand whether ONPs will be used for harm reduction by smokers and SLT users in Appalachia, a region with that is disparately impacted by tobacco use.

This study qualitatively examined perceptions of ONPs, including perceived risks and perceived substitutability for cigarettes and SLT, and perceptions of ONP marketing in Appalachian adults who smoke cigarettes and use SLT. Our study was guided by a framework characterizing the appeal of novel tobacco and nicotine products among consumers [21]. This framework posits that consumer perceptions and uptake of novel products are influenced by features of the product and its marketing. Importantly, tobacco product features and product marketing are potential targets of policy and regulation, depending on how they influence tobacco use behavior and related public health outcomes. This qualitative investigation was an initial step to understand how Appalachian adults perceive ONPs to guide future research that can inform policy.

## Methods

### Design

We conducted a series of focus groups for a qualitative descriptive study. We based reporting on the COREQ [22] checklist for qualitative research (S1 Appendix). Focus groups are an established data collection method in qualitative research using interactive discussions to gather participants’ experiences, opinions, beliefs, perceptions, and attitudes [23].

### Participants and procedures

In February-May of 2022, we conducted 10 focus groups with cigarette smokers and SLT (chew, snuff, dip, snus) users aged ≥21 years who resided in Ohio Appalachian counties [24]. We used purposive recruitment through social media advertising, institutional study registries, and word of mouth referrals. Participants completed an online screener capturing age, tobacco use, and contact information. Eligible participants were those who were able to complete study procedures in English, smoked ≥5 cigarettes per day (smokers) or used SLT at least once per day for the past 30 days, and used other tobacco/non-therapeutic nicotine ≤ 10 days per month.

We reviewed screener responses for data quality (e.g., potential duplicate responses) and eligibility and contacted eligible participants to schedule an online focus group. Before the focus group, participants received a secure web link to complete informed consent and an online survey capturing sociodemographics, smoking and SLT use history, other tobacco/nicotine use, and awareness and trial of ONPs [25]. We stratified focus groups by participants’ use of cigarettes and SLT. If participants met eligibility criteria for cigarette smoking and SLT use, we scheduled their focus group based on the product they used more often.

The focus groups were conducted virtually and moderated by the first (woman) or second (man) author with the other co-facilitating to assist with technical assistance. Both are trained facilitators with relevant academic training (master/doctoral degree) and certification (e.g., Certified Clinical Research Professional), and experience in relevant research fields (e.g., public health, nicotine and tobacco research). The principal investigator (last author) trained study personnel, supervised focus groups, and reviewed the recordings and transcripts to give feedback on moderation/facilitation. We based the number of focus groups on saturation in thematic coding [26]. At the beginning of each focus group, the facilitator provided a description and purpose of the study prior to introducing the researchers present and their roles for the discussion and posing open-ended questions using a semi-structured focus group guide. The facilitator used clarifying questions and probing when necessary to elucidate or expound on certain topics and to keep the focus on the topic at hand.

We developed a semi-structured focus group guide to elicit participants’ experiences, opinions, and risk perceptions of harm and addiction of ONPs, substitutability of ONPs for cigarettes/SLT, and reactions to example ONP marketing (S2 Appendix). Content of the focus group guide was grounded in an overarching framework characterizing the appeal of novel tobacco and nicotine products among consumers. According to this framework, characteristics of the product, how consumers perceive the product (e.g., perceived health risks, perceived addictiveness, perceived substitutability for other products), and how they respond to product marketing are key factors that affect uptake and use of novel products [21]. We developed the focus group guide to elicit participants’ perspectives on these key factors that are likely to influence ONP uptake and use. We identified prominent ONP brands from the literature [13] and obtained example marketing from a tobacco advertising database [27, 28] and online searches. We used visual aids for the discussion, including images of ONPs and example marketing materials (S2 Appendix). Focus groups lasted about one hour and used audio and video digital recording to collect data. We reviewed transcripts automatically generated by the recording software against recordings and edited for accuracy. Transcripts were not returned to participants for comment or correction. Participants received a $50 gift card. All participants completed informed consent online and The Ohio State University Institutional Review Board approved all procedures (2021C0187).

### Data analysis

We conducted analyses using NVivo 12. We used an iterative thematic approach to focus group data by applying simultaneous inductive and deductive analysis as focus groups were conducted [23]. We chose this analytic approach because it is not tied to a specific epistemological or theoretical perspective, and it enabled us to iteratively revisit and refine our coding scheme as we conducted the focus groups [29]. Two coders developed initial codes based on the discussion guide and codes were continually refined as new transcripts were coded (e.g., adding codes for new themes that emerged) [30]. We then used the initial set of codes to develop a focused coding scheme comprising themes capturing important groups of initial codes. Both coders independently developed theme codes, and any conflicts were resolved through consensus by discussion with a third coder. This process ensured adequate agreement between the two coders (kappas > 0.70).

We used the coding scheme to review data from our initial coding of all transcripts, organize the data into prominent categories containing recurring themes, and identify the most prominent themes across all transcripts. We continued recruitment until no new themes emerged. For the results, we selected illustrative quotes based on their depiction of prominent themes.

### Methodological rigor

We used a systematic approach to our data collection and analysis to ensure methodological rigor. This included steps to ensure data quality described above (e.g., carefully screening participants for eligibility, reviewing transcripts against recordings for accuracy, double coding all transcripts, arbitrating coding discrepancies with a third coder), and additional measures recommended in the literature [31]. Our team comprises experts in nicotine and tobacco research (including oral nicotine and tobacco products) and researchers with expertise in qualitative methods. We used peer debriefing with experts on our team who were not involved in conducting the focus groups or coding the data to develop the focus group guide based on the framework that informed our work [21], review our initial codebook, review our focused coding scheme, and reach consensus on illustrative quotes reported as examples of coded data. Coders and the full team engaged in data triangulation based on recent quantitative survey [25] data to enrich our analysis and the validity of our findings. Finally, we evaluated data that appear to disconfirm our primary patterns and themes (i.e., negative case analysis) and we revised our inferences and conclusions accordingly [23].

## Results

### Sample characteristics

Overall, 207 participants completed screening, 167 were eligible (80.7%), and 37 (22.2% of those eligible) completed procedures. We were unable to contact most participants who screened eligible but did not compete procedures (100/130), others were scheduled to participate but did not, cancelled before focus groups, or had scheduling conflicts. Table 1 shows the sample characteristics. We conducted 5 focus groups with smokers (range 2–5 participants) and 5 with SLT users (range 2–6 participants). Categories and themes that emerged from the focus groups are described below, with exemplary quotes in S1 Table.

**Table 1:** Study sample characteristics.

	Current Smoker	Current SLT User	Overall
	(N = 19)	(N = 18)	(N = 37)
**Age** Mean (SD)	45.9 (10.8)	38.7 (10.1)	42.4 (10.9)
**Gender**			
Female	14 (73.7%)	0 (0%)	14 (37.8%)
Male	5 (26.3%)	18 (100%)	23 (62.2%)
**Race**			
White	18 (94.7%)	18 (100%)	36 (97.3%)
Non-white	1 (5.3%)	0 (0%)	1 (2.7%)
**Ethnicity**			
Hispanic	0 (0%)	1 (5.6%)	1 (2.7%)
Non-Hispanic	19 (100%)	17 (94.4%)	36 (97.3%)
**Education**			
Less than college	17 (89.5%)	10 (55.6%)	27 (73.0%)
College degree or higher	2 (10.5%)	8 (44.4%)	10 (27.0%)
**Employment status**			
Employed Part Time/Full Time	8 (42.1%)	17 (94.4%)	25 (67.6%)
Not Currently Employed	11 (57.9%)	1 (5.6%)	12 (32.4%)
**Household income**			
$50,000 or less	13 (68.4%)	4 (22.2%)	17 (46%)
$50,001 to $75,000	1 (5.3%)		1 (2.7%)
Greater than $75,000	3 (15.8%)	12 (66.7%)	15 (40.5%)
Prefer not to say	2 (10.5%)	2 (11.1%)	4 (10.8%)
**Cigarettes per day** Mean (SD)	16.3 (5.8)		
**SLT uses per day** Mean (SD)		8.61 (6.52)	
**Past year quit attempt**			
No	13 (68.4%)	14 (77.8%)	27 (73.0%)
Yes	6 (31.6%)	4 (22.2%)	10 (27.0%)
**Oral nicotine pouch awareness**			
No	9 (47.4%)	1 (5.6%)	10 (27.0%)
Yes	10 (52.6%)	17 (94.4%)	27 (73.0%)
**Oral nicotine pouch ever use**			
No	5 (26.3%)	4 (22.2%)	9 (24.3%)
Yes	5 (26.3%)	13 (72.2%)	18 (48.6%)
**Oral Nicotine pouch current use**			
No	3 (15.8%)	7 (38.9%)	11 (29.7%)
Yes	2 (10.6%)	6 (33.3%)	8 (21.6%)

### Theme: Perceived risks of ONPs

#### Sub-theme: Health risks (Table 2)

**Table 2:** Exemplary quotes for sub-themes of perceived risks of ONPs.

**Oral and gastrointestinal health**: Oral or gastrointestinal health risks associated with ONPs. *“… But I figure the health risks can’t be worse than smoking*, *because at least I’ll be able to breathe*. *Probably end up with mouth or stomach cancer because it is still nicotine and chemicals*.*”* (Female, age 39, cigarette smoker, has used ONPs) *“I think you kind of with like the nicotine pouches you probably run a similar risk of like plaque and gum issues*, *esoph-*, *esophageal issues*, *stomach issues*, *stuff like that*, *so I imagine if you swallow it and don’t spit it out and keep doing that and doing that*, *it’s probably gonna have a similar effect than like tobacco would just because of like how you handle it*.*”* (Male, age 41, smokeless tobacco [SLT] user, has used ONPs)
**Cardiovascular and lung health**: Cardiovascular or respiratory risk associated with ONPs. *“I think they would be not*, *not as harmful to your health just because you’re not inhaling smoke with nicotine*, *I feel they would be safer that’s just my opinion*.*”* (Female, age 56, cigarette smoker, has used ONPs) *“I would say*, *definitely with your lungs*. *I mean that’s number one*, *and you know just the lungs*. *I’m sure that it’s still going to increase the mess with your heart*, *a little bit with the nicotine*, *but mainly just the lungs…be a great way to start quitting*, *though*.*”* (Male, age 37, cigarette smoker, never used ONPs)
**Cancer**: Cancer risks associated with ONPs. *“I’m sure there’s more cancerous things going on with the real snuff*, *you know your chances are better doing the*, *you know*, *the fake pouches or whatever*.*”* (Male, age 44, SLT user, has used ONPs)
**Youth appeal and initiation**: Risks associated with youth appeal and initiation of ONPs. *“I would think possibly younger children would have access to it*, *because if it’s in your mouth nobody’s going to see it so you’re going to have younger people starting so their risk of addiction is easier*.*”* (Female, age 46, cigarette smoker, never used ONPs)
**Addiction risk**: Addiction risks of ONPs. “*I mean the addiction is to the nicotine itself and habit*, *I mean I think habit becomes more of an addiction than the nicotine itself*.” (Male, age 32, SLT user, has used ONPs)

* Refer to S1 Table for additional quotes

The most frequently discussed health risks included oral, gastrointestinal, cardiopulmonary, and cancer risks. Most smokers perceived ONPs had a similar risk to cigarettes but were concerned about exchanging one health risk for another, emphasizing decreased respiratory health risk but increased oral and gastrointestinal risk. In contrast, SLT users tended to view ONPs having similar or less risk than SLT but did not reference exchanging risks.

*“Your health risks are just transferred from your respiratory system to your*, *you know*, *your esophageal*, *mouth*, *you know*, *area*, *as far as that goes*. *Yeah*, *you might be able to breathe a little bit better but*, *you know your tongue might have issues or your gums your teeth […]”*

(Male, age 42, cigarette smoker, never used ONPs)

*“I’m sure there’s more cancerous things going on with the real snuff*, *you know your chances are better doing the*, *you know*, *the fake pouches…”*

(Male, age 44, SLT user, has used ONPs)

#### Sub-theme: Youth appeal and initiation risk (Table 2)

Participants viewed ONPs to be appealing to youth due to the ability to conceal use and use ONPs where other tobacco is discouraged or prohibited:

*“I know I’m one of the people who started in school […] we were in a shop [class]*, *who cares*, *but the ones sitting in class all day and stuff like that*. *They’re gonna want to do something like that*, *if they can’t smoke*, *or even hit their e-cigarette or something like that*, *it’s*, *it’s an easy alternative*.*”*

(Male, age 36, SLT user, never used ONPs)

Participants also cited packaging as potentially appealing to youth:

*“The packages of the nicotine pouches*, *they kind of attract like younger people*, *like they’re brighter*, *like they’re new*, *it’s like come and try me*, *you know what I mean*.*”*

(Male, age 33, SLT user, never used ONPs)

#### Sub-theme: Addiction risk (Table 2)

Participants perceived ONPs to be as or more addictive than their usual tobacco product:

*“Yeah*, *nicotine is addictive*, *because you can use it so discreetly and just wherever […]”*

(Female, age 23, cigarette smoker, never used ONPs)

However, participants differentiated between addiction to nicotine and habit or routine:

“*I mean the addiction is to the nicotine itself and habit*, *I mean I think habit becomes more of an addiction than the nicotine itself*.”

(Male, age 32, SLT user, has used ONPs)

Similarly, another participant emphasized the complexity of this addiction, explaining:

*“I think anytime you deal with nicotine here you put yourself at risk for addiction*. *[…] It’s not only the thing that you get addicted to*, *the nicotine*, *you just get addicted to the*, *the routine like after*, *after I eat*, *I put chew in*, *when I wake up*, *I put chew in*, *before I go to bed*, *I put chew in*. *It’s just part of life*.*”*

(Male, age 33, SLT user, never used ONPs)

### Theme: Substitutability of ONPs for cigarettes/SLT

#### Sub-theme: Substitutability for cigarettes/SLT (Table 3)

**Table 3:** Exemplar quotes for sub-themes of substitutability of ONPs for cigarettes/SLT.

**Substitutability for cigarettes**: Ability to substitute ONPs for cigarettes. *“I like the fact that I didn’t have to smoke to get my nicotine and I just can’t see myself using them on a regular basis*. *For*, *I don’t know*, *it just seems inconvenient*, *I guess*, *I enjoy my cigarette too much to supplement it*, *you know with the nicotine pouch*.*”* (Female, age 56, cigarette smoker, has used ONPs) “*I’m a smoker and I wouldn’t use them*. *Because I just*, *I don’t*, *I don’t think I’ll be able to tolerate that thing in my mouth*.” (Female, age 54, cigarette smoker, never used ONPs)
**Substitutability for SLT**: Ability to substitute ONPs for SLT. *“Yeah*, *I mean I feel like you could probably use them interchangeably whenever you*, *whenever you’d want*. *You’re going to still have your old timers that that will never use them ever because they’re stuck in their ways*, *but I’m sure most times wherever you could put a chew in […] you could put a nicotine pouch in and I’m sure they could be used interchangeably*. *Depends on the person*.*”* (Male, age 33, SLT user, never used ONPs)
**Situational Substitutability**: Using ONPs when using usual tobacco product is not allowed. *“Anyway*, *I’ve used the Rogue*. *And I’ve also used the Zyn and a lot of it*, *some places that you know I normally can’t use my Copenhagen snuff*. *You know ball games*. *Different you know different scenarios like that*. *Or working at my office*. *You know*.*”* (Male, age 44, SLT user, has used ONPs) *“Yeah I used*, *it’s been a couple years*, *but when we were traveling and flying on an airplane obviously you can’t smoke in an airport or on an airplane so I used those to get my fix while we were traveling*.*”* (Female, age 56, cigarette smoker, has used ONPs)
**Cessation aid**: ONPs to reduce or quit tobacco use. *“I wouldn’t supplement cigarettes just and then do nicotine pouches for the rest of my life*, *though it’s either quit or nothing at all it’s either quit or still smoke*. *So I guess it’s more of a quitting tool I pointed as*.*”* (Male, age 37, cigarette smoker, never used ONPs)

* Refer to S1 Table for additional quotes

Most smokers perceived ONPs as situational rather than complete substitutes for cigarettes:

*“I like the fact that I didn’t have to smoke to get my nicotine and I just can’t see myself using them on a regular basis*. *For*, *I don’t know*, *it just seems inconvenient*, *I guess*, *I enjoy my cigarette too much to supplement it*, *you know with the nicotine pouch*.*”*

(Female, age 56, cigarette smoker, has used ONPs)

Participants also reported ONP characteristics, such as discreteness, may make them acceptable for use in places where smoking is prohibited or discouraged:

“*Because you can use it so discreetly and just wherever […] I know*, *personally*, *if I use them*, *I could use them at work*, *while I’m just driving with kids in the car*, *and the store*, *out in public areas where normally I couldn’t smoke*.”

(Female, age 23, current smoker, never used ONPs)

Similarly, SLT users perceived ONP use as situational substitutes but with notable distinctions. For instance, the similar mode of delivery between ONPs and SLT made them more open to try ONPs:

“*I’d be open to try it but I’m*, *you know*, *I never really dipped for the taste*.*”*

(Male, age 25, SLT user, never used ONPs)

Additional appealing characteristics of ONPs, such as being cleaner and more socially acceptable than SLT, made them more suitable for situational use:

*“If I can dip*, *I’m going to dip*. *I know I bought a couple of cans of On*! *and […] let’s say you already brushed your teeth*, *or whatever*, *and you don’t want to have to deal with that again and getting your mouth all nasty […] Or if it’s*, *you know*, *if you’re indoors and*, *you know*, *you can’t spit or whatever*. *So*, *it’s situations where you know*, *I*, *it’s I could dip but it’s easier to*, *you know*, *just use a nicotine pouch*.*”*

(Male, age 31, SLT user, has used ONPs)

Smokers and SLT users noted similarities and differences between their usual product and ONPs, and the lack of satisfaction that made them perceive ONPs to be a situational substitute:

“*Most smokers would rely on both [cigarettes and ONPs] to get through their day or their evening or whatever the situation is that would require the use of both*, *instead of smoking*, *I think*, *for the most part*, *most smokers tend to enjoy smoking just a little bit*. *Be it the hand*, *the mouth*, *or the taste*, *or whatever it is*.”

(Male, age 42, cigarette smoker, never used ONPs)

Some participants rejected ONPs due to perceived differences: “*I’m a smoker and I wouldn’t use them*. *Because I just*, *I don’t*, *I don’t think I’ll be able to tolerate that thing in my mouth*,” (Female, age 54, cigarette smoker, never used ONPs) while others indicated they would rather “*quit nicotine altogether*” (Female, age 56, cigarette smoker, has used ONPs) than switch to ONPs.

#### Sub-theme: Cessation (Table 3)

Most smokers and SLT users perceived ONPs to be an option for reducing or quitting tobacco. For instance, a SLT user noted:

*“I think it would probably be easier […] than like the gum or the gum or the patches or anything like that*. *Like he said… it’s more natural like it’s*, *it’s more natural to us […]*.*”*

(Male, age 33, SLT user, never used ONPs)

Similarly, a smoker stated:

*“I wouldn’t supplement cigarettes just and then do nicotine pouches for the rest of my life*, *though*. *It’s either quit or nothing at all*, *it’s either quit or still smoke*. *So*, *I guess it’s more of a quitting tool […]”*

(Male, age 37, cigarette smoker, never used ONPs)

A current smoker and ONP user found them helpful to reduce cigarette smoking and withdrawal symptoms:

*“Once you get used to ’em*, *I like ‘em*. *And they do help to quit smoking*. *And I try and tell my friends that and they were given away for free all the time*, *so I started giving them to people that were talking about quitting smoking…because like I watched my dad die*, *I watched my mom die*, *I know smoking’s bad*. *I know I can’t breathe…I can’t run*. *And at least this keeps me from getting angry from not smoking*.*”*

(Female, age 39, cigarette smoker, has used ONPs)

Experience with nicotine replacement therapy (NRT) also influenced their perceptions:

“*I’ve tried*, *the patches*. *Those left like a burn on my skin*, *and that itched and burned real bad*, *so I don’t want to do those*. *And I tried the gum and those tasted absolutely horrible*. *This [nicotine pouches] is awesome because they taste good and I don’t have to spit ‘em*. *The coffee tastes [like] I’m drinking…it’s not a strong flavor*, *but it’s just enough to make me happy and it tastes like coffee*. *They’re fun*.*”*

(Female, age 39, cigarette smoker, has used ONPs)

Similarly, experience (or lack thereof) with e-cigarettes impacted how participants compared ONPs to e-cigarettes. For example, most SLT users indicated they:

“*Wouldn’t even consider it [e-cigarette]*. *I think most snuff chewers*, *smokeless tobacco chewers*, *you know*, *I don’t think they would go to […] the e-cigarettes […]*”

(Male, age 44, SLT user, has used ONPs)

While smokers who had negative experiences with e-cigarettes preferred ONPs, those who had positive experiences preferred e-cigarettes due to the similarities with cigarette smoking (see S1 Table). Another factor influencing how participants compared ONPs to NRT or e-cigarettes was price and availability of coupons and discounts (see S1 Table).

### Theme: Perceptions of ONP marketing

#### Sub-theme: Marketing content (Table 4)

**Table 4:** Exemplar quotes for sub-themes of reactions to example ONP marketing materials.

**Marketing content**: Appearance and first impressions of ONP Marketing. *“I think the colors make you want to read it*. *It’s appealing to the eye*,*”* (Male, age 53, SLT user, has used ONPs) *“I mean I don’t*, *I never had coffee snuffs [sic] so coffee nicotine pouches is kind of attractive*,*”* (Male, age 33, SLT user, never used ONPs) *“It only comes in three and six milligrams*,*”* (Male, age 29, SLT user, has used ONPs) *“Cigarettes are always in a pack all lined up nice and neat and the rows and the way they’re in the display case they’re lined up nice and neat and their rows it just kind of clicks in your mind*,*”* (Male, age 60, cigarette smoker, has used ONPs) *“Unless you are in the market for it*, *you’re not even looking at it*.*”* (Male, age 37, SLT user, has used ONPs)
**Target audience**: Intended audience of ONP marketing. *“No*, *I mean this looks like a more New York style city type to me*. *Not Appalachia Ohio*.*”* (Male, Age 44, SLT user, Has used ONPs) *“I dislike that it looks like candy and maybe younger generations may be attracted to it*, *because it looks like it’s a candy*.*”* (Female, Age 61, cigarette smoker, never used ONPs) *“I think this is more targeted to you know people that haven’t used any kind of tobacco products before as a gateway*, *so to speak*, *into tobacco products*. *It seems more like it’s for a new user versus switching someone from tobacco products to*.*”* (Male, age 28, SLT user, has used ONPs) *“If you want to quit and that’s the stuff you’re looking for*, *you’re going to be looking at this stuff*. *If you really don’t have any interest in quitting then you’re not even paying any attention*.*”* (Male, age 37, SLT user, has used ONPs)

* Refer to S1 Table for additional quotes

Participants drew from experience with cigarettes and SLT to formulate ONP marketing perceptions. Marketing content that drew participants’ attention included color, flavor, nicotine strength, packaging, and “tobacco-free” claims. For example: *“I think the colors make you want to read it*,*”* (Male, age 53, SLT user, has used ONPs)*“I never had coffee snuffs [sic] so coffee nicotine pouches is kind of attractive*,*”* (Male, age 33, SLT user, never used ONPs) *“It only comes in three and six milligrams*,*”* (Male, age 29, SLT user, has used ONPs) *“Cigarettes are always in a pack all lined up nice and neat and the rows and the way [the pouches]they’re in the display case they’re lined up nice and neat and their rows it just kind of clicks in your mind*,*”* (Male, Age 60, cigarette smoker, has used ONPs) and *“you know it’s got these things on the top*, *where it says ‘chew and spit free no combustion tobacco free odor free’ that would entice me more to at least investigate the product[…]”*(Female, age 39, cigarette smoker, never used ONPs). Participants also indicated ads were *“soft*, *clean ad*, *it’s gonna make everything seem soft and clean*,*”* (Female, age 36, cigarette smoker, never used ONPs) lacked brand-recognition: *“It just doesn’t seem to have like an edge that you want*, *like the reputation*, *the branding*, *it’s like a dentist office*,*”* (Male, age 25, SLT user, never used ONPs) and appeared like “*chewing gum*” (Male, Age 36, cigarette smoker, has used ONPs) or a “*tin of mints*” (Female, Age 59, cigarette smoker, never used ONPs).

Despite some appealing features, participants expressed they would “*overlook*” (Male, age 50, SLT user, never used ONPs and Male age 55, SLT user, never used ONPs) ONP ads and “*Unless you are in the market for it*, *you’re not even looking at it*” (Male, age 37, SLT user, has used ONPs). While more information about ONPs was appealing, ads with the most text and images led to disinterest and were perceived to be like medical information.

#### Sub-theme: Target audience (Table 4)

Participants surmised ONP ads would appeal to several target audiences, most prominently youth and new users:

*“I think this is more targeted to*, *you know*, *people that haven’t used any kind of tobacco products before as a gateway*, *so to speak*, *into tobacco products*. *It seems more like it’s for a new user versus switching someone from tobacco products […]”*

(Male, age 28, SLT user, has used ONPs).

Other groups identified as targets of the ONP ads included urban populations, high socioeconomic status groups, and tobacco users seeking to cut down or quit.

“*No*, *I mean this looks like a more New York style city type to me*. *Not Appalachia Ohio*,”

(Male, age 44, SLT user, has used ONPs)

*“I don’t feel like this product’s for me I don’t feel like I’m high class enough*, *you know*, *like drinking beer on Saturdays not martinis with James Bond or something I don’t feel like this is in my class*,*”*

(Male, age 28, SLT user, has used ONPs)

*“It looks like a yuppie ad*, *that is what it looks like to me*. *Because anybody here that’s chews skoal or loose leaf or anything like that*, *one of the things that it seems to be the same characteristic for all of them*, *and some type of a cowboy thing to it*. *That’s one thing I noticed*, *it’s really yuppie looking*.*”*

(Male, age 53, SLT user, has used ONPs)

## Discussion

We qualitatively examined Ohio Appalachian tobacco users’ perceptions of ONPs and their marketing. ONPs are new to the U.S. market, and limited available evidence indicates ONPs may carry a lower toxicant burden than cigarettes or SLT [18] and thus represent a potential for reduced harm for adult tobacco users who switch to ONPs. Appalachia is disproportionately impacted by tobacco use and experiences disparities in related health outcomes. Our findings add to the literature by qualitatively characterizing Appalachian adult tobacco users’ perceptions of ONPs and their marketing.

Recent research indicates the majority of young adult combustible and noncombustible tobacco users who had never used ONPs were uncertain whether ONPs are less harmful than cigarettes or e-cigarettes [17]. In our sample, smokers and SLT users viewed ONPs to be as addictive as cigarettes/SLT and better suited for situational use because they are “cleaner,” more socially acceptable, and can be used discreetly when cigarettes/SLT are prohibited or not acceptable. Situational substitution could exacerbate disparities in Appalachia by maintaining or increasing nicotine dependence if ONPs are used in settings when one would typically abstain from tobacco. However, switching completely from cigarettes/SLT to ONPs could reduce disparities in tobacco-related health outcomes affecting Appalachia. Perceptions on switching to ONPs varied between smokers and SLT users, by knowledge and experience of alternatives (e.g., e-cigarettes, NRT), and by motivation to quit. High cost was cited as a limiting factor for NRT relative to ONPs, which present a more affordable option at $4 or less per can in Ohio. Several participants discussed their experience using ONPs to cut down or quit tobacco, and others acknowledged ONPs’ potential as a quitting tool but cited lack of motivation to quit as a governing factor. Clinical studies have shown adult smokers find ONPs to be more satisfying than NRT, increased nicotine bioavailability than NRT, and shown ONPs perform similar to NRT at relieving withdrawal symptoms [32, 33]. Our findings highlight aspects of ONPs that are likely to be important to adult tobacco users’ ONP uptake and use patterns, but research is needed to understand potential health risks of ONPs, and how adult smokers and SLT users will use them (e.g., as situational or complete substitutes).

ONP marketing will be critical to whether adult cigarette smokers and SLT users try ONPs. ONP marketing emphasizes features such as nicotine content and co-promotes ONPs with cigarettes to target cigarette smokers [10–12, 34–39]. There is limited research on the effects of ONP marketing, including which features may impact the appeal of ONPs in adult tobacco users. In our study, ONP marketing elicited limited appeal overall among smokers and SLT users. However, colors, flavors, nicotine concentration, and “tobacco-free” claims were discussed as advertising features that may lead people to try ONPs. Tobacco and nicotine product marketing and novel product features are potential targets of policy and regulation, depending on how they influence perceptions and behavior. Further research on how the features of ONP marketing affect tobacco users and non-users is needed to inform such measures.

Study participants highlighted the potential appeal of ONPs to young people across themes. ONP features such as discrete use, colorful packaging, and advertising were thought to be appealing to youth and new users. Although research is limited, there is emerging evidence on the potential appeal of ONPs in young people. A 2020 study of young adults found willingness to use ONPs was significantly higher among tobacco users (combustible, non-combustible, and dual) than non-users and approximately half of the participants across all groups were uncertain about the relative harm of ONPs compared to cigarettes and e-cigarettes [17]. A 2021 study found oral nicotine products (including ONPs) were among the most popular non-combustible tobacco products among high school aged youth [40]. ONP use was found to be disproportionately greater for Hispanic, sexual minority, gender minority, and female youth, and those who used combustible and non-combustible tobacco [40]. This aligns with tobacco companies’ efforts to expand marketing of non-combustible products to such demographic groups [41], and with our findings that adult tobacco users perceived ONPs may appeal to such demographics. Research is needed to understand ONP appeal among populations such as youth, non-users, and minority groups. This will advance understanding of the impact of ONPs on public health and inform policy and regulatory approaches to ONPs.

Our findings should be interpreted considering limitations of the study. We conducted the study with a convenience sample of adult tobacco users from a region affected by tobacco use disparities. Our methodological approach and research on sample sizes in qualitative studies [26] supports our finding that we reached thematic saturation but given the characteristics of our sample and our focus on a specific population, generalizability of the findings to broader populations is limited. To understand the appeal of ONPs in other populations, more research is needed. Due to the COVID-19 pandemic, we conducted focus groups virtually incorporating recommendations from the literature [42, 43] such as using smaller group sizes. While the virtual format may have limited participation to those with internet access, it reduced some barriers to participation (e.g., the need for travel) and provided a platform to facilitate candid dialogue. We experienced few technical problems affecting participation and participant feedback was favorable, but this limitation may affect generalizability of our findings.

## Conclusions

The study findings indicate adult tobacco users perceived ONPs to have similar or less risks of health harm relative to other tobacco products and they viewed ONPs to be similarly addictive. Due to characteristics such as the ability to conceal ONP use, participants viewed ONPs to be appealing to youth. The findings also indicate ONPs primarily appeal to adult tobacco users as situational substitutes, but some may use them to cut down or quit other tobacco use. With respect to marketing, participants were most drawn to branding in ONP marketing, but content emphasizing nicotine (e.g., nicotine concentration, “tobacco free” nicotine claims) was also prominently discussed. These marketing claims have the potential to convey lower risks [20]. These findings highlight potential ways in which ONPs and their marketing may appeal to adult tobacco users and other groups. Research to monitor ONP uptake, patterns of use relative to other tobacco products, and marketing strategies will be critical to understand how ONPs may impact public health, and how they can be appropriately addressed through policy and regulation.

## Supporting information

S1 Appendix COREQ checklist. (PDF)

S2 Appendix Focus group guide. (PDF)

S1 Table Exemplar quotes for major categories and themes. (DOCX)
