# Supplementary material for: Perceptions of oral nicotine pouches & their marketing among Ohio Appalachia smokers and smokeless tobacco users
Source: PLoS One. 2023 Oct 30;18(10):e0293597. doi: 10.1371/journal.pone.0293597 (PMC10615305; doi:10.1371/journal.pone.0293597)
Supplement: S2 Appendix — (PDF) [file pone.0293597.s002.pdf]

Supplementary Appendix 1

Focus Group Guide

Overview

Welcome, my name is [Facilitator Name] and we thank you for taking the time to join us today. As a reminder, we are conducting this focus group as part of a study led by researchers at the Ohio State University Comprehensive Cancer Center to understand people's thoughts about tobacco and nicotine products, reasons they may or may not use these products, and whether or not the products appeal to them. We will discuss more details of these topics shortly, but to start I want to review some information for our group today.

First, my role as the facilitator is to introduce the discussion topics and ask questions to promote discussion. Although I am here to help with the discussion, our goal with the focus groups is to hear from you about your thoughts and opinions and for you to have a conversation as a group. [Note taker name] is also here to take notes during the discussion to make sure we capture everything that is said.

Next, I would like remind you that we will use preferred first names only during the discussion. Anything you say as part of the discussion is completely confidential. The researchers will not include any individuals' identifying information, such as their names, with statements made during the discussion. We will be audio recording the discussion to make sure we capture everything that is said. All notes from the group and the audio recording will be stored securely at the Ohio State University Comprehensive Cancer Center. Nothing that you say during the discussion will be linked to identifying information about you, such as your name. We hope this encourages you to speak openly.

During our discussion you can use the controls in Zoom when needed. We ask that you please keep your audio and your video camera on so we can see and hear you as much as possible. If you need to turn off your camera or mute your audio for any reason, at the bottom left of your screen there are controls to do this. For example, if you are in a setting where there may be background noise and that could make it difficult for others to hear, you can put yourself on mute as needed. There is also an option to "raise hand" on the bottom right of your screen (for some computers and phones, this appears when you click on "reactions"). You can use this as needed to get our attention if you have something to add to the discussion. But we encourage you to speak up to participate in our discussion.

Finally, we would like to briefly introduce everyone in the group to start our discussion. My name is [Facilitator], and I will be the group facilitator today.

My name is [Note Taker] and I will be taking notes during the discussion today.

[Allow participants to introduce themselves, call on participants' as needed to get started]

What questions do you have before we begin? [Allow participants to ask questions]

Introduction to Nicotine Pouches

We want to learn more about what you know about a product called nicotine pouches, and your thoughts about nicotine pouches. Nicotine pouches usually come in cans or tins containing 15 to 20 pouches. The pouches are usually white or light brown and they are placed under your lip to deliver nicotine through the gums. Unlike snus and other forms of smokeless tobacco such as chew or dip, nicotine pouches do not contain tobacco. The picture we are showing on the screen is an example of what nicotine pouches look like. Some common brands include Zyn, Velo, On!, and Rogue.

Image of Oral Nicotine Pouches in a white circular plastic container. Container lid and branding was not present in the image shown to participants.

What have you seen or heard about nicotine pouches before this study?

Probes: Where did you see or hear this information?
Before today, have you heard of specific nicotine pouch brands?

Have you ever tried nicotine pouches?
If yes: Can you tell me about your first time trying them? For example, what was going on at the time? What made you decide to try them?

How do you use nicotine pouches now? For example, how often? Do you use them with other substances?

What did you like about nicotine pouches? What did you not like?

Probe: What brands do you remember trying? What did you like about these brands? What did you not like?

Probe: What flavors do you remember trying? What did you like about these flavors? What did you not like?

Probe: What different nicotine "strength" pouches have you tried? What made you choose to try these nicotine strengths? What did you like about these nicotine strengths? What did you not like?

Probe: How did the nicotine pouches compare to your usual brand of cigarettes [smokeless tobacco]?

For those who have not tried nicotine pouches, what would make you want to try them?

Probe: What makes you not want to try nicotine pouches?

Perceived Risks and Benefits

We are interested in learning whether you have seen or heard anything about the possible health risks, or health benefits, of using nicotine pouches.

First, let's talk about the possible health risks of nicotine pouches. What have you heard about possible health risks of nicotine pouches?

> *Probe: Where did you see or hear this information?*
> *Probe, if have not heard of anything: What types of health risks do you think nicotine pouches may cause for people who use them?*

Have you heard anything about how the health risks of nicotine pouches compare to cigarettes (or smokeless tobacco)?

> *Probe, if have not heard anything: How do you think the health risks of nicotine pouches might compare to cigarettes (or smokeless tobacco)?*

What have you heard about the potential for nicotine pouches to lead to addiction, or the inability to quit using them?

> *Probe: Where did you see or hear this information?*
> *Probe, if have not heard of anything: What do you think about the risk of addiction to nicotine pouches for people who use them?*

Have you heard anything about how the risk of addiction to nicotine pouches compares to cigarettes (or smokeless tobacco)?

> *Probe, if have not heard anything: How do you think the risk of addiction to nicotine pouches might compare to cigarettes (or smokeless tobacco)?*

Now, let's talk about the possible health benefits of nicotine pouches. What have you heard about possible health benefits of nicotine pouches?

> *Probe: Where did you see or hear this information?*
> *Probe, if have not heard of anything: What do you think might be the health benefits of nicotine pouches for people who use them?*

Perceived Substitutability

Thank you. We are also interested in your thoughts about whether you can use nicotine pouches instead of smoking cigarettes [using smokeless tobacco, like chew or dip].

To what extent do you think smokers [smokeless tobacco users] could use nicotine pouches in situations when they cannot smoke cigarettes [use smokeless tobacco], such as places where smoking [using smokeless tobacco, or spitting] is not allowed?

Probe: What makes you think this way?

What do you think about switching completely from smoking cigarettes [using smokeless tobacco] to using nicotine pouches? By switching completely, I mean completely stopping smoking cigarettes [using smokeless tobacco] and using nicotine pouches instead.

Probe: What makes you think this way?

Probe: Are there reasons that you think it would be easier or harder to switch completely to nicotine pouches than other methods for quitting, like nicotine gum or lozenge, the nicotine patch, or a nicotine inhaler?

Probe: Do you think it would be easier or harder to switch completely to nicotine pouches than switching completely to an e-cigarette, like JUUL?

To what extent would you consider nicotine pouches to be a tobacco product, like cigarettes [smokeless tobacco]?

Probe: What makes you consider or not consider nicotine pouches to be a tobacco product like cigarettes [smokeless tobacco]?

Perceptions of Marketing

Next we are going to show some example advertisements for nicotine pouches. We would like to hear what you think and feel about the advertisements. These are examples of nicotine pouch advertisements that have appeared in places such as magazines, websites, emails, and at stores such as gas stations and convenience stores.

Ad # 1

Ad images are available by request from the corresponding author.

What about this ad grabs your attention most?

What do you like or dislike about this ad?

Have you seen advertisements such as these before?
Probe: Where?
Probe: Do you recall any reactions you had to the advertisement(s) then?

Did or would an advertisement such as this prompt you to buy nicotine pouches?
Probe: What aspects of an advertisement such as this would prompt you to buy or not buy nicotine pouches?

Would you say advertisements such as these make nicotine pouches appealing or unappealing to smokers [smokeless tobacco users]?
Probe: What aspects of advertisements such as these make nicotine pouches appealing or unappealing?

Ad # 2

Ad images are available by request from the corresponding author.

What about this ad grabs your attention most?

What do you like or dislike about this ad?

Have you seen advertisements such as these before?

Probe: Where?

Probe: Do you recall any reactions you had to the advertisement(s) then?

Did or would an advertisement such as this prompt you to buy nicotine pouches?

Probe: What aspects of an advertisement such as this would prompt you to buy or not buy nicotine pouches?

Would you say advertisements such as these make nicotine pouches appealing or unappealing to smokers [smokeless tobacco users]?

Probe: What aspects of advertisements such as these make nicotine pouches appealing or unappealing?

Ad # 3

Ad images are available by request from the corresponding author.

What about this ad grabs your attention most?

What do you like or dislike about this ad?

Have you seen advertisements such as these before?
Probe: Where?
Probe: Do you recall any reactions you had to the advertisement(s) then?

Did or would an advertisement such as this prompt you to buy nicotine pouches?
Probe: What aspects of an advertisement such as this would prompt you to buy or not buy nicotine pouches?

Would you say advertisements such as these make nicotine pouches appealing or unappealing to smokers [smokeless tobacco users]?
Probe: What aspects of advertisements such as these make nicotine pouches appealing or unappealing?

Close

Does anyone have anything else they would like to bring up about nicotine pouches or the topics we discussed?

Thank you. That concludes the questions we have for you today.

A member of our team will be in touch with you soon about any next steps in the study, and to send your gift card for participating in the study. Thank you again for your time today.
