# Supplementary material for: Perceptions of oral nicotine pouches & their marketing among Ohio Appalachia smokers and smokeless tobacco users
Source: PLoS One. 2023 Oct 30;18(10):e0293597. doi: 10.1371/journal.pone.0293597 (PMC10615305; doi:10.1371/journal.pone.0293597)
Supplement: S1 Table — (DOCX) [file pone.0293597.s003.docx]

**S1 Table**. Exemplar quotes for major categories and themes

| Perceived Risks of Oral Nicotine Pouches (ONPs) |
| --- |
| **A. Oral and gastrointestinal health:** Oral or gastrointestinal health risks associated with ONPs.   - *“I did notice when I, when I use them more often…and I always do the same corner…it almost gets like a sensitive sensation there. But I figure the health risks can’t be worse than smoking, because at least I’ll be able to breathe. Probably end up with mouth or stomach cancer because it is still nicotine and chemicals.”* (Female, age 39, cigarette smoker, has used ONPs) - *“I think you kind of with like the nicotine pouches you probably run a similar risk of like plaque and gum issues, esoph-, esophageal issues, stomach issues, stuff like that, so I imagine if you swallow it and don’t spit it out and keep doing that and doing that, it’s probably gonna have a similar effect than like tobacco would just because of like how you handle it.”* (Male, age 41, smokeless tobacco [SLT] user, has used ONPs) - *“Your health risks are just transferred from your respiratory system to your, you know, your esophageal, mouth, you know, area, as far as that goes. Yeah, you might be able to breathe a little bit better but, you know your tongue might have issues or your gums your teeth, or whatever else, and I know a lot of people are pretty self-conscious about how they look nobody's looking at your lungs, people are looking at your mouth, teeth, and face.”* (Male, age 42, cigarette smoker, never used ONPs) |
| **B. Cardiovascular and lung health:** Cardiovascular or respiratory risk associated with ONPs.   - *“I think they would be not, not as harmful to your health just because you’re not inhaling smoke with nicotine, I feel they would be safer that’s just my opinion.”* (Female, age 56, cigarette smoker, has used ONPs) - *“I would say, definitely with your lungs. I mean that's number one, and you know just the lungs. I’m sure that it's still going to increase the mess with your heart, a little bit with the nicotine, but mainly just the lungs…be a great way to start quitting, though.”* (Male, age 37, cigarette smoker, never used ONPs) - *“…but I really think you’re just trading one evil for the other because the health benefits of your mouth your teeth and all that are gonna be worse.”* (Female, age 59, cigarette smoker, never used ONPs) |
| **C. Cancer:** Cancer risks associated with ONPs.   - *“I’m sure there’s more cancerous things going on with the real snuff, you know your chances are better doing the, you know, the fake pouches or whatever.”* (Male, age 44, SLT user, has used ONPs) - *“Probably like risk of cancer, you know mouth and gum cancer, same things that go along with rubbing regular chew. Just a little bit, slightly better.”* (Male, age 33, SLT user, never used ONPs) - *“I wish there was more information on the side effects of it. Am I gonna get stomach cancer? Probably, but I will with smoking too. I’ll be able to breathe if I actually quit, quit smoking.”* (Female, age 39, cigarette smoker, has used ONPs) |
| **D. Youth appeal and initiation:** Risks associated with youth appeal and initiation of ONPs.   - *“I think it would cause a lot more younger smokers too, younger people taking to nicotine because that’s something they can hide from their parents and everybody.” Similarly, another participant stated that “I would think possibly younger children would have access to it, because if it’s in your mouth nobody’s going to see it so you’re going to have younger people starting so their risk of addiction is easier.”* (Female, age 46, cigarette smoker, never used ONPs) - *“The packages of the nicotine pouches, they kind of attract like younger people like they’re brighter, like they’re new it's like come and try me, you know what I mean.”* (Male, age 33, SLT user, never used ONPs) - *“I know I’m one of the people who started in school […] we were in a shop, who cares, but the ones sitting in class all day and stuff like that. They’re gonna want to do something like that, if they can’t smoke, or even hit their e-cigarette or something like that, it’s, it’s an easy alternative.”* (Male, age 36, SLT user, never used ONPs) |
| **E. Addiction risk**: Addiction risks of ONPs.   - “*I mean the addiction is to the nicotine itself and habit, I mean I think habit becomes more of an addiction than the nicotine itself*.” (Male, age 32, SLT user, has used ONPs) - *“I think anytime you deal with nicotine here you put yourself at risk for addiction. I started rubbing snuff like, like I said, probably the first snuff I ever did I was probably eight, eight and a half years old. I was as sick as a dog. Then I started I did it started doing it again like 10, 10 and a half, 11. I’ve done it every day since so. It's not only the thing that you get addicted to the nicotine you just get addicted to the, the routine like after, after I eat I put chew in when I wake up, I put chew in, before I go to bed, I put chew in. It’s just part of life.”* (Male, age 33, SLT user, never used ONPs) - *“Yeah, nicotine is addictive, because you can use it so discreetly and just wherever […]”* (Female, age 23, cigarette smoker, never used ONPs) |
| Substitutability of ONPs for cigarettes/SLT |
| **F. Substitutability for cigarettes**: Ability to substitute ONPs for cigarettes.   - *“I like the fact that I didn't have to smoke to get my nicotine and I just can't see myself using them on a regular basis. For, I don't know, it just seems inconvenient, I guess, I enjoy my cigarette too much to supplement it, you know with the nicotine pouch.”* (Female, age 56, cigarette smoker, has used ONPs) - *“I honestly don't think I could […] trade out my cigarettes for nicotine pouches. Because I, I would use them more as a quitting tool as well just trying to get off of nicotine, all together, but I don't I don't see myself using them in the long-term situation.”* (Female, age 56, cigarette smoker, has used ONPs) - “*Because you can use it so discreetly and just wherever you, I mean, I know, personally, if I use them, I could use them at work, while I’m just driving with kids in the car, and the store, out in public areas where normally I couldn’t smoke*.” (Female, age 23, cigarette smoker, never used ONPs) - “*Most smokers would rely on both to get through their day or their evening or whatever the situation is that would require the use of both, instead of smoking, I think, for the most part, most smokers tend to enjoy smoking just a little bit. Be it the hand, the mouth, or the taste, or whatever it is*.” (Male, age 42, cigarette smoker, never used ONPs) - “*I’m a smoker and I wouldn't use them. Because I just, I don't, I don't think I’ll be able to tolerate that thing in my mouth.*” (Female, age 54, cigarette smoker, never used ONPs) |
| **G. Substitutability for SLT**: Ability to substitute ONPs for SLT.   - *“Yeah, I mean I feel like you could probably use them interchangeably whenever you, whenever you’d want. You’re going to still have your old timers that that will never use them ever because they’re stuck in their ways, but I’m sure most times wherever you could put a chew in […] you could put a nicotine pouch in and I’m sure they could be used interchangeably. Depends on the person.”* (Male, age 33, SLT user, never used ONPs) - *“If I can dip, I’m going to dip. I know I bought a couple of cans of On! and […] let’s say you already brushed your teeth, or whatever, and you don’t want to have to deal with that again and getting your mouth all nasty, and just being able to put one […] Or if it’s, you know, if you’re indoors and, you know, you can’t spit or whatever. So it’s situations where you know, I, it’s I could dip but it’s easier to, you know, just use a nicotine pouch.”* (Male, age 31, SLT user, has used ONPs) |
| **H. Situational Substitutability**: Using ONPs when using usual tobacco product is not allowed.   - *“Anyway, I've used the Rogue. And I’ve also used the Zyn and a lot of it, some places that you know I normally can't use my Copenhagen snuff. You know ball games. Different you know different scenarios like that. Or working at my office. You know.”* (Male, age 44, SLT user, has used ONPs) - *“Yeah I used, it’s been a couple years, but when we were traveling and flying on an airplane obviously you can’t smoke in an airport or on an airplane so I used those to get my fix while we were traveling.”* (Female, age 56, cigarette smoker, has used ONPs) - *“I’ll be honest, I never really thought about the health risk. And the fact that I would substitute for smoking for a situation…for situational use… so you know. I don’t think as a smoker I don’t think about, you know, what the health risk is of situational use of something to make sure that I’m not yelling at flight attendants on nine hours flights.”* (Female, age 39, cigarette smoker, has used ONPs) |
| **I. Cessation aid:** ONPs to reduce or quit tobacco use.   - *“I think it would probably be easier, it's easier method, than, like the gum or the gum or the patches or anything like that. Like he said… it's more natural like it's, it's more natural to us whether, whether it would really help me or not I don't know because it's like I’m, I’m just so used to doing the same thing over and over and over and it's I mean, I would be willing to try it, but I would have about a 50% success, I would assume a 50% success.”* (Male, age 33, SLT user, never used ONPs) - *“I wouldn’t supplement cigarettes just and then do nicotine pouches for the rest of my life, though it’s either quit or nothing at all it’s either quit or still smoke. So I guess it’s more of a quitting tool I pointed as.”* (Male, age 37, cigarette smoker, never used ONPs) - *“Once you get used to ’em, I like ’em. And they do help to quit smoking. And I try and tell my friends that and they were given away for free all the time, so I started giving them to people that were talking about quitting smoking…because like I watched my dad die, I watched my mom die, I know smoking’s bad. I know I can't breathe…I can’t run. And, at least this keeps me from getting angry from not smoking.”* (Female, age 39, cigarette smoker, has used ONPs) - “*I've tried, the patches. Those left like a burn on my skin, and that itched and burned real bad, so I don’t want to do those. And I tried the gum and those tasted absolutely horrible. This [nicotine pouches] is awesome because they taste good and I don’t have to spit‘em. The coffee tastes [like] I’m drinking…it's not a strong flavor, but it's just enough to make me happy and it tastes like coffee. They're fun.”* (Female, age 39, cigarette smoker, has used ONPs) - *“I would [try nicotine pouches] too, but I think mine would be because of the cost, you know, they would, they, depending on the price of them now, they would more than likely be more cost effective than the other reason or the other items that you had mentioned [nicotine replacement therapy], because the other items are quite expensive.”* (Female, age 56, cigarette smoker, has used ONPs) |
| Reactions to example ONP marketing materials |
| **J. Marketing content**: Appearance and first impressions of ONP Marketing.   - *“I think the colors make you want to read it. It's appealing to the eye,”* (Male, age 53, SLT user, has used ONPs) - *“I mean I don’t, I never had coffee snuffs [sic] so coffee nicotine pouches is kind of attractive,”* (Male, age 33, SLT user, never used ONPs) - *“It only comes in three and six milligrams,”* (Male, age 29, SLT user, has used ONPs) - *“Cigarettes are always in a pack all lined up nice and neat and the rows and the way they're in the display case they're lined up nice and neat and their rows it just kind of clicks in your mind,”* (Male, age 60, cigarette smoker, has used ONPs) - *“You know it's got these things on the top, where it says ‘chew and spit free no combustion tobacco free odor free’ that would entice me more to at least investigate the product[…]”* (Female, age 39, cigarette smoker, never used ONPs) - *“Soft, clean ad, it's gonna make everything seem soft and clean,”* (Female, age 36, cigarette smoker, never used ONPs) - *“It just doesn't seem to have like an edge that you want, like the reputation, the branding, it’s like a dentist office,”* (Male, Age 25, SLT user, Never used ONPs) and appeared like *“chewing gum”* (Male, Age 36, Current smoker, Has used ONPs) or a *“tin of mints.”* (Female, age 59, cigarette smoker, never used ONPs) - *“Unless you are in the market for it, you’re not even looking at it.”* (Male, age 37, SLT user, has used ONPs) |
| **K. Target audience**: Intended audience of ONP marketing.   - *“No, I mean this looks like a more New York style city type to me. Not Appalachia Ohio.”* (Male, Age 44, SLT user, Has used ONPs) - *“I don't feel like this product’s for me. I don't feel like I’m high class enough, you know, like drinking beer on Saturdays not martinis with James Bond or something I don't feel like this is in my class.”* (Male, age 28, SLT user, has used ONPs) - *“I dislike that it looks like candy and maybe younger generations may be attracted to it, because it looks like it’s a candy.”* (Female, Age 61, cigarette smoker, never used ONPs) - *“I think this is more targeted to you know people that haven't used any kind of tobacco products before as a gateway, so to speak, into tobacco products. It seems more like it's for a new user versus switching someone from tobacco products to.”* (Male, age 28, SLT user, has used ONPs) - *“If you want to quit and that’s the stuff you’re looking for, you’re going to be looking at this stuff. If you really don’t have any interest in quitting then you’re not even paying any attention.”* (Male, age 37, SLT user, has used ONPs) - *“It looks like a yuppie ad, that is what it looks like to me. Because anybody here that's chews skoal or loose leaf or anything like that one of the things that it seems to be the same characteristic for all of them, and some type of a cowboy thing to it. That's one thing I noticed, it’s really yuppie looking.”* (Male, age 53, SLT user, has used ONPs) |
